# Multidisciplinary Treatment of Non-Spine Bone Metastases: Results of a Modified Delphi Consensus Process

**DOI:** 10.1016/j.ctro.2022.04.009

**Published:** 2022-04-26

**Authors:** Erin F. Gillespie, Noah J. Mathis, Max Vaynrub, Ernesto Santos Martin, Rupesh Kotecha, Joseph Panoff, Andrew L. Salner, Alyson F. McIntosh, Ranju Gupta, Amitabh Gulati, Divya Yerramilli, Amy J. Xu, Meredith Bartelstein, David M. Guttmann, Yoshiya J. Yamada, Diana Lin, Kaitlyn Lapen, Deborah Korenstein, David G. Pfister, Allison Lipitz-Snyderman, Jonathan T. Yang

**Affiliations:** a Department of Radiation Oncology, Memorial Sloan Kettering Cancer Center, New York, NY, USA; b Center for Health Policy and Outcomes, Memorial Sloan Kettering Cancer Center, New York, NY, USA; c Department of Orthopaedic Surgery, Memorial Sloan Kettering Cancer Center, New York, NY, USA; d Department of Interventional Radiology, Memorial Sloan Kettering Cancer Center, New York, NY, USA; e Department of Radiation Oncology, Miami Cancer Center, Miami, FL, USA; f Department of Radiation Oncology, Hartford Healthcare, Hartford, CT, USA; g Department of Radiation Oncology, Lehigh Valley Cancer Institute, Lehigh Valley Health Network, Allentown, PA, USA; h Department of Hematology & Medical Oncology, Lehigh Valley Cancer Institute, Lehigh Valley Health Network, Allentown, PA, USA; i Department of Anesthesiology & Critical Care, Memorial Sloan Kettering Cancer Center, New York, NY, USA; j Department of Medicine, Memorial Sloan Kettering Cancer Center, New York, NY, USA

**Keywords:** Bone Metastases, Oligometastases, SBRT, Radiofrequency ablation, Cryoablation, Pathologic fracture

## Abstract

**Purpose:**

Local treatment for bone metastases is becoming increasingly complex. National guidelines traditionally focus only on radiation therapy (RT), leaving a gap in clinical decision support resources available to clinicians. The objective of this study was to reach expert consensus regarding multidisciplinary management of non-spine bone metastases, which would facilitate standardizing treatment within an academic-community partnership.

**Methods and Materials:**

A multidisciplinary panel of physicians treating metastatic disease across the Memorial Sloan Kettering (MSK) Cancer Alliance, including community-based partner sites, was convened. Clinical questions rated of high importance in the management of non-spine bone metastases were identified via survey. A literature review was conducted, and panel physicians drafted initial recommendation statements. Consensus was gathered on recommendation statements through a modified Delphi process from a full panel of 17 physicians from radiation oncology, orthopaedic surgery, medical oncology, interventional radiology, and anesthesia pain. Consensus was defined a priori as 75% of respondents indicating “agree” or “strongly agree” with the consensus statement. Strength of Recommendation Taxonomy was employed to assign evidence strength for each statement.

**Results:**

Seventeen clinical questions were identified, of which 11 (65%) were selected for the consensus process. Consensus was reached for 16 of 17 answer statements (94%), of which 12 were approved after Round 1 and additional 4 approved after Round 2 of the modified Delphi voting process. Topics included indications for referral to surgery or interventional radiology, radiation fractionation and appropriate use of stereotactic approaches, and the handling of systemic therapies during radiation. Evidence strength was most commonly C (n = 7), followed by B (n = 5) and A (n = 3).

**Conclusions:**

Consensus among a multidisciplinary panel of community and academic physicians treating non-spine bone metastases was feasible. Recommendations will assist clinicians and potentially provide measures to reduce variation across diverse practice settings. Findings highlight areas for further research such as pathologic fracture risk estimation, pre-operative radiation, and percutaneous ablation.

## Highlights

• Evidence is emerging for new paradigms in the management of non-spine bone metastases.
• Consensus was feasible amongst physicians in both academic and community-based practice settings.
• Topics deemed of highest importance for consensus included referral for surgical stabilization and approach to *peri*-operative radiation, preferred radiation fractionation and appropriate use of stereotactic techniques, and clinical scenarios classified as potentially “complex” warranting multidisciplinary discussion.

## Introduction

Bone metastases are common among patients with metastatic disease, and often cause pain and functional impairment [1]. While comprehensive multidisciplinary guidelines exist for spinal metastases [2], national guidelines for the management of non-spine bone metastases have traditionally focused on radiation therapy (RT) to palliate pain, and surgical intervention and bone modifying agents to treat or prevent pathologic fractures [3], [4]. A recent review showed the most common locations for these lesions include the hip/pelvis, ribs, shoulder, and femur and comprise 46% of all bone metastases treated in a tertiary radiation oncology center [5]. However, as systemic therapies have evolved and options for local therapy have increased, the utility of existing guidelines in routine care is increasingly limited. With this growing complexity, variation in practice patterns by clinical setting has been observed, demonstrating possible opportunities to standardize practice and improve outcomes [6].

In this study, we leveraged a community-academic partnership (in which the main academic center has a dedicated multidisciplinary team for managing bone metastases) to develop consensus recommendations that build on existing guidelines and would be applicable in multiple practice settings. We hypothesized that consensus on key multidisciplinary topics in the management of non-spine bone metastases would be feasible and provide the foundation for a future prospective study aimed to improve patient access to high-quality, patient-centered metastatic cancer care across diverse practice settings.

## Methods

### Consensus clinical setting and panel composition

The Memorial Sloan Kettering (MSK) Cancer Alliance is a community-academic partnership between MSK (a tertiary academic center) and community-based hospitals (Hartford HealthCare Cancer Institute, Lehigh Valley Cancer Institute, and Miami Cancer Institute at Baptist Health South Florida). MSK founded this Alliance with the goals of a) rapidly bringing the newest standards of care into community-based cancer practice settings and b) increasing patients’ access to clinical trials in their local areas. MSK is unique in having radiation oncologists specializing in metastatic disease and co-leading both multidisciplinary clinics and a weekly tumor board [7].

**Fig. 1:**
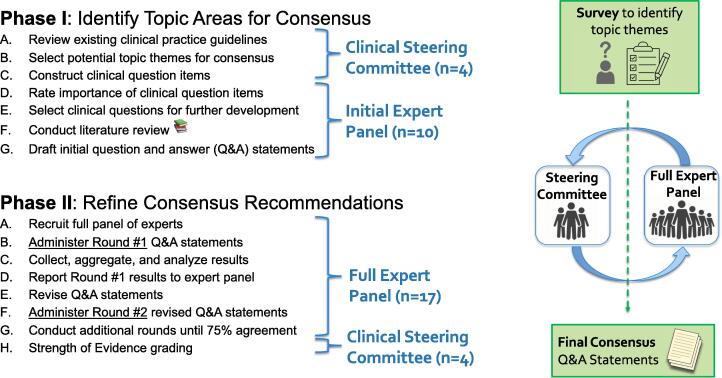
Schematic of the consensus process. 2 rounds of the modified Delphi process were required to reach consensus on 15 question/answer statements.

The current study involved clinicians from multiple disciplines managing patients with non-spine bone metastases across the community-academic partnership. The *clinical steering committee* was comprised of 2 radiation oncologists, one orthopaedic surgeon with fellowship training in orthopaedic oncology, and one interventional radiologist, all from the main academic center. The *initial expert panel* (for Phase 1 to identify topics for consensus) was comprised of 7 radiation oncologists, 2 orthopaedic surgeons with fellowship training in orthopaedic oncology, and one interventional radiologist and represented the MSK Cancer Alliance community-based partners. The *full expert panel* (for Phase 2 consensus voting) consisted of 9 radiation oncologists, 2 surgeons with fellowship training in orthopaedic oncology, 2 medical oncologists, one interventional radiologist specializing in oncologic procedures, one anesthesia pain specialist, and one medical physicist. Full panel participation by practice location included: MSK (n = 11), Miami Cancer Institute at Baptist Health South Florida (n = 2), Lehigh Valley Cancer Institute (n = 2), and Hartford HealthCare Cancer Institute (n = 1).

### Consensus process

We conducted a modified Delphi consensus process between March 2020 and March 2021. This iterative process leverages a systematic progression of repeated rounds of voting to determine expert group consensus where high-level evidence may be lacking and opinion is important [8]. We incorporated specific Delphi procedures including de-identified response, iteration and controlled feedback, and summary statistics to elicit and refine group judgement, in keeping with established methods [9]. Modifications to the Delphi included group meetings at the end of each Round (as documented previously through “Estimate-Talk-Estimate” [10] and pre-specifying the recommendation agreement threshold. (See Fig. 1 for a process schema). We outline a 2-phase process for identifying topics then refining consensus recommendations, similar to a prior modified Delphi [11].

### Phase 1: Identify topic areas for consensus

The clinical steering committee first conducted a review of existing clinical practice guidelines to identify potential gaps in available recommendations. Common clinical questions warranting multidisciplinary input in the management of non-spine bone metastases were identified. An online survey was distributed to multidisciplinary physicians across the MSK Cancer Alliance, requesting respondents rank the relative importance of each question on a 5-point Likert scale from “Not important” to “Essential.” (See Supplemental Survey for instrument) The results of this survey were summarized by the clinical steering committee and presented to the initial expert panel in a meeting held on Zoom. Questions were selected for further development based on importance rating by panel participants and perceived feasibility of reaching consensus. Those with a median score of 5 (“Essential”) were automatically advanced to the next step of the consensus process.

A literature review included references in PubMed published since January 1, 2010. Search terms specific to each previously identified question were drafted, using both MeSH terms and text (Supplemental Text). Exclusion criteria were non-human studies, publication in a language other than English, and studies conducted using subjects 18 years of age or younger. Based on these search terms, 1498 published articles were identified. Titles were screened (NM and KL) for exclusion criteria and relevance to the questions. A total of 90 primary articles were identified and summarized in tables that were then provided to 1–2 assigned expert(s) from the panel would drafted initial consensus answer statements (See Supplemental Draft Statement for an example clinical question and answer statement worksheet with evidence summary). Initial drafts were reviewed by the clinical steering committee who also had to approve any proposed modifications to the questions (including additional questions).

### Phase 2: Refine consensus recommendations and grade evidence strength

Once preliminary question and answer statements (recommendations) were drafted, the full expert panel was recruited. We initiated Round 1 of voting by the full expert panel regarding their level of agreement or disagreement with each preliminary answer statements using a 5-point Likert scale from “strongly disagree” to “strongly agree,” and were asked to provide comments. Voting was done individually via an online survey and results were de-identified. Modifications were proposed by the steering committee after each round of voting and presented to the panel in a Zoom meeting. Round 2 voting proceeded and the process continued until 75% of panel members “agreed” or “strongly agreed” with the statement, which was specified a priori.

The Strength of Recommendation Taxonomy (SORT) was employed to grade evidence strength [12]. The literature supporting the consensus recommendations was reviewed in duplicate and strength of recommendation was assigned (EFG, JTY), with any discrepancies between the two reviewers resolved by a third (NM).

## Results

In Phase 1, 17 clinical questions were identified, of which 5 questions (29%) obtained a median importance score of “essential” and were automatically advanced to the next phase of consensus. These questions addressed complex case scenarios warranting multidisciplinary discussion, indications for surgical stabilization and *peri*-operative radiation fields, and radiation fractionation and indications for Stereotactic body radiation therapy (SBRT) (See Supplemental Table for details). The initial expert panel selected additional questions such that 11 total clinical questions (59%) proceeded for further development. One question regarding radiation margins was omitted due to recent publication of an international survey on the topic [13]. In the drafting stage, the following changes were approved by the steering committee and resulted in a final list of 12 questions: 1) one question added with approval by the steering committee: “How important is the determination of treatment intent for patients undergoing local therapy for non-spine bone metastases?”; 2) Two questions regarding radiation fractionation (pain and oligometastases) were collapsed into a single question; 3) Interventional radiology techniques were split into 2 questions.

In Phase 2, 17 total physicians were recruited (as described in Methods). Answer statements were drafted and amended in response to Round 1 voting and feedback from the full panel, including splitting the systemic therapy recommendations into 5 separate statements for voting, for a total of 16 statements. Twelve of 17 (71%) statements were approved in Round 1 with the pre-specified 75% threshold for agreement, and 4 statements (24%) were approved in Round 2. Consensus could not be reached on standardizing normal organs contoured for radiation avoidance. The final set of clinical questions and answer statements are presented in Table 1. Evidence strength was most commonly rated as C (n = 7), followed by B (n = 5), and A (n = 3). A narrative summary of the evidence supporting each consensus recommendation is presented here.

**Table 1:** Question and answer statements for each clinical question in the treatment of non-spine bone metastases. % Agreement represents the percentage of study group members indicating “Agree” or “Strongly Agree” with the statement.

Question	Answer	% Agreement	Strength of Recommendation
1. How important is the determination of treatment intent for patients undergoing local therapy for non-spine bone metastases?	All patients undergoing local therapy for non-spine bone metastases should have treatment intent, generally defined as either symptom management and/or tumor control, specified at the time of initial consultation.	100%	B
2. What tool should be used to estimate performance status in the setting of metastatic disease?	All patients being treated for non-spine bone metastases should have, at minimum, Karnofsky Performance Status (KPS) or ECOG, determined and documented at time of initial consultation.	100%	A
3. How are uncomplicated bone metastases defined, and what is the preferred treatment for symptomatic uncomplicated bone metastases?	A. Uncomplicated bone metastases can be defined as: painful bone metastases unassociated with impending or existing pathologic fracture or existing spinal cord or cauda equina compression, and which have not had prior local therapy. B. Radiation is first-line treatment for symptomatic uncomplicated bone metastases.	88%	A
4. What clinical scenarios of non-spine bone metastases can be classified as “complex,” and should be considered for review by a multidisciplinary team?	Clinical scenarios that can be classified as “complex” and therefore warrant review by a multidisciplinary team include, but are not limited to: Pathologic fracture or impending fracture Recurrence after initial local therapy to metastatic lesion Difficulty determining origin of a patient’s pain (i.e. multiple lesions, defining mechanical vs. biological pain) Oligometastatic disease (defined as <5 metastatic lesions) in a patient with >6 months prognosis and stable disease after first-line systemic therapy	94%	C
5. When should a patient with non-spine bone metastases be referred to surgery for prophylactic surgical fixation/stabilization?	Referral to a surgeon should be considered if the patient’s medical status and oncologic life expectancy are permissive of surgery and **any** of the following are present: 1. Lytic long bone or pelvic lesion with pain that is worsened with activity, 2. Any significant lesion in the femur that is either lytic or painful, 3. Progressive growth after radiation, or 4. Failure of palliation with radiation	88%	B
6. When should referral to interventional radiology for image-guided stabilization (such as cementoplasty) be considered for a patient with non-spine bone metastases?	Percutaneous *stabilization* (e.g. cementoplasty, cemented screw fixation) should be considered for patients who have been evaluated by orthopaedic surgery and are not surgical candidates if the following are met: 1. The patient has mechanical pain (pain that is worsened by weight-bearing, positional changes, or activity) 4 or more weeks after radiation. 2. Metastatic lesion is in the pelvis or the epiphysis/metaphysis of a long bone with intact subchondral bone. There is insufficient evidence to guide the choice between various stabilization modalities or whether ablation and stabilization should be used concurrently.	88%	C
7. When should referral to interventional radiology for image-guided ablation be considered for a patient with non-spine bone metastases?	Percutaneous *ablation* (e.g. cryoablation, radiofrequency ablation, microwave ablation) should be considered for patients with symptomatic bone metastases if all of the following are met: 1. First-line radiation has not provided adequate pain relief (evaluated at least 4 weeks after treatment) and reirradiation is not preferred; 2. Metastatic lesion is in the pelvis, a long bone, the sternum, or the scapula; 3. Bone is not at risk for fracture; 4. Pain is non-mechanical; 5. Ablation target is at least 1 cm from functional neurologic elements, visceral organs, and joint surfaces. There is insufficient evidence to guide the choice between various ablation modalities or whether ablation and stabilization should be used concurrently.	81%	B
8. What radiation regimens are preferred for symptomatic uncomplicated non-spine bone metastases?	Most radiation treatments can and should be delivered in 5 or fewer fractions, regardless of technique (2D vs 3D vs SBRT).	100%	A
9. When should stereotactic radiation (SBRT/SABR) be considered for the treatment of non-spine bone metastases?	• For symptomatic patients, stereotactic radiation can be considered for those with high KPS (70+) and radioresistant histology, or in the setting of re-treatment when more conformal therapy is needed to avoid exceeding dose constraints. • For asymptomatic patients with oligometastatic disease, phase II randomized data for SABR is promising, although enrollment on a clinical trial is preferred until phase III data is available.	94%	B
10. What is the preferred approach to radiotherapy in the setting of stabilization surgery for non-spine bone metastases?	After stabilization surgery, coverage of the entire orthopaedic hardware within the radiation field is recommended to reduce local recurrence. There is insufficient evidence to recommend the use of pre-operative radiation therapy to a non-spine bone metastasis outside of a clinical trial.	81%	B
11.1 When should systemic therapies be held for patients undergoing radiation therapy for non-spine bone metastases?	Immunotherapy and hormone therapies (excluding hormone-based chemotherapy) are generally **considered safe and can be continued during radiation. **Potential exception may include radiation to organs that have had immunotherapy related complications.	94%	B
11.2 When should systemic therapies be held for patients undergoing radiation therapy for non-spine bone metastases?	For patients on VEGF and VEGFR inhibitors (i.e. bevacizumab), radiation fields that involve the bowel may increase the risk of bowel injury. However, due to the unclear duration of this risk before and after VEGF or VEGFR inhibitor dosing, as well as the long half-lives of antiangiogenic drugs, for select patients it may be reasonable to offer RT during or soon after administration of such agents. **Patients should be counselled on these risks prior to RT, and subsequent use of VEGF or VEGFR inhibitors after RT is discouraged.**	88%	C
11.3 When should systemic therapies be held for patients undergoing radiation therapy for non-spine bone metastases?	With regard to BRAF inhibitors (dabrafenib, vemurafenib, encorafenib) and MEK inhibitors, we defer to the 2016 ECOG guidelines, which recommend holding for at least 3 days before and after RT due to risk for skin toxicity.	100%	C
11.4 When should systemic therapies be held for patients undergoing radiation therapy for non-spine bone metastases?	When systemic agents have **known toxicities to organs that overlap** with the radiation field (e.g. doxorubicin with sternal metastases), consider holding systemic therapy for 2 half-lives.	94%	C
11.5 When should systemic therapies be held for patients undergoing radiation therapy for non-spine bone metastases?	For CDK inhibitors and other targeted therapies and cytotoxic agents, there is insufficient evidence to guide the decision on when to hold systemic therapy. Most palliative radiation trials defer to the treating physician’s preference. **We hold these agents for 1-2 half-lives before radiation treatment, and for 24 hours after radiation.**	88%	C

### Topic 1: Treatment intent

In the context of treatment for metastatic disease, the understanding of prognosis is important to how patients make decisions about future treatments and enrollment in hospice [14], [15]. However, patients and family members’ understanding of treatment intent and prognosis in the setting of advanced cancer is often inaccurate [16], [17], [18], [19]. To ensure that patients have the information necessary to make informed decisions, treatment intent ought to be clearly specified when initial recommendations are made for patients with bone metastases.

### Topic 2: Reporting of performance status

Performance status has been shown in both retrospective and prospective studies to correlate with survival for patients with advanced cancer [20], [21], [22], [23], [24]. In one study, Karnofsky Performance Status (KPS) alone was shown to have a stronger correlation with survival than clinical predictions made by oncologists [25], though this result has not been consistent [26]. The performance status scale proposed by the Eastern Cooperative Oncology Group (ECOG) has also been consistently shown to correlate with survival [27].

### Topic 3: Definition and treatment of uncomplicated bone metastases

A definition of uncomplicated bone metastases has previously been proposed to include lesions which are painful, and unassociated with impending or existing pathologic fracture, and unassociated with existing spinal cord or cauda equina compression [28]. In addition to the complicated lesions identified previously, we chose to consider metastases with prior local therapy as complicated, as there are often different treatment considerations in the recurrent setting.

Radiation therapy is effective in alleviating pain for 60% of individuals with bone metastases and provides complete pain relief for about 25% of patients, with low rates of toxicity [29]. First-line RT is supported by high-level evidence from the meta-analysis of multiple randomized controlled trials and consensus guidelines from multiple professional organizations [3], [30], [31].

### Topic 4: Indications for multidisciplinary review

A retrospective analysis of patients presented at the MSK non-spine bone metastases tumor board over a 3-month period (n = 42) revealed that 83% of the patients were presented for the four reasons listed in Table 1. Multidisciplinary discussion is also beneficial for individuals with symptoms that may or may not be caused by metastases, when there is uncertainty over the origin of a patient’s pain or whether it is mechanical in nature. Lastly, while metastasis-directed radiation therapy for oligometastases has a growing body of evidence, the definition and indications continue to evolve. The multidisciplinary setting allows for additional input regarding patient selection, timing with systemic therapy, and radiation dose.

### Topic 5: Indications for surgical referral

Multiple retrospective studies have demonstrated that prophylactic stabilization of the femur may be associated with improved survival compared to surgery for a completed femur fracture [32], [33]. Regardless, clinicians should aim to promptly refer for surgical evaluation those patients at imminent risk of pathologic long bone fracture, particularly in the femur, given known risk of morbidity and mortality caused by femur fracture [34].

Readily available morphology-based scoring tools for predicting pathologic long bone fracture have limited accuracy [35], [36]. Nonetheless, a review by Siegel et al. points out that the presence of pain with weight bearing is likely the most important factor in determining risk for pathologic fracture [37]. Extent of cortical involvement and osteolytic lesions have also been shown to be associated with higher risk of pathologic fracture [38], [39].

### Topic 6: Considerations for percutaneous stabilization

Percutaneous stabilization techniques were introduced for patients with metastatic disease in the spine (e.g. vertebroplasty), but more recently have been used for individuals with non-spine bone metastases. Retrospective data have shown these procedures to provide short-term palliation for patients who have persistent pain despite RT and are not candidates for surgery [40], [41]. One single-arm prospective study in which 50 patients with non-spine bone metastases were treated with cementoplasty after failure of conventional therapy reported durable pain relief with 9 months of follow-up, but two patients with lesions in the femoral diaphysis suffered femur fractures, [42] prompting our recommendation for consideration of cementoplasty only for lesions in the epiphysis or metaphysis of a long bone. Broader reviews of the data for percutaneous stabilization exist, though these are generally limited by heterogeneous groups of patients and difficulty separating the effects of the percutaneous stabilization from other treatments [43], [44], [45]. Therefore, we reserve these techniques for patients in whom traditional treatments are contraindicated or ineffective.

### Topic 7: Considerations for percutaneous ablation

As stated above, radiation therapy is an effective first-line treatment for alleviation of pain in patients with non-spine bone metastases. However up to 40% of patients will not achieve pain relief after first-line RT [29]. For these individuals, retreatment with radiation therapy is generally safe and provides pain relief in about half of cases [46], [47]. However in some cases reirradiation may prove ineffective or challenging due to adjacent normal tissue dose constraints or a patient’s inability to tolerate simulation and treatment. In these situations, percutaneous ablation provides an alternative.

Goetz et. al. conducted a single-arm prospective study in which patients who had failed standard therapy and been seen by a radiation oncologist were treated with radiofrequency ablation, and experienced clinically significant pain relief up to 24 weeks after the procedure [48]. Other studies of percutaneous ablation have been done, but have heterogeneous patient populations and often use ablation in combination with cementoplasty, making conclusions about the effectiveness of ablation difficult [40], [43]. Patients who are at high risk of pathologic fracture are not good candidates for percutaneous ablation and should be referred to an orthopaedic surgeon.

### Topic 8: Radiation treatment for symptomatic uncomplicated bone metastases

Multiple prospective randomized trials have demonstrated that single fraction regimens are as effective as multi-fraction regimens for symptom relief for bone metastases [49], [50], [51], [52], [53]. However, single fraction regimens are more likely to require retreatment, which has been shown to have limited use in routine practice [54]. A small randomized trial demonstrated equivalence between 5- and 10-fraction regimens in terms of pain relief and time to retreatment [55]. Meanwhile, trials have shown safety and efficacy of stereotactic treatments delivered in 1–5 fractions [56], [57], and preliminary data suggests reductions in financial toxicity with shorter SBRT regimens for metastases [58]. It is therefore important to minimize the burden of treatment visits for patients with metastatic disease, and we recommend treatment in no more than 5 fractions for those with symptomatic, uncomplicated non-spine bone lesions.

### Topic 9: Considerations for stereotactic radiation

In the palliative setting, a phase II randomized controlled trial showed single fraction SBRT to be non-inferior to multi-fraction conventional RT, with higher rates of pain response in those receiving SBRT [57]. We favor using conventional RT for bone metastases in most cases, with SBRT reserved for patients at high risk for recurrence, in particular those with radioresistant tumors or prolonged expected survival [5].

Phase II data for the use of ablative radiation for patients with oligometastases are promising [59], [60], with the strongest evidence in patients with lung cancer [59], however patient selection is likely important and therefore such treatments remain largely investigational and presentation to a multidisciplinary team (as noted above) or enrollment in a clinical trial is preferred.

### Topic 10: Combining surgery and radiation

Multiple retrospective analyses have shown that rates of local recurrence are lower for patients who receive RT to the entire length of orthopaedic hardware after stabilization surgery [61], [62]. Pre-operative radiation to non-spine bone metastases is an area warranting further research, with a prospective clinical trial underway.

### Topic 11: Holding systemic therapy during radiation

Immunotherapy and hormone therapy agents are most likely safe to administer during radiation, although limitations in the data still exist. Phase I trials have shown that concurrent immunotherapy and RT is safe, and the effectiveness of this combination may be promising for certain patients [63], [64]. Hormonal therapy is also safe when used concurrently with RT, as demonstrated by several large retrospective analyses in the primary breast cancer population which did not show meaningful safety concerns [65], [66].

The safety of VEGF or VEGFR inhibiting drugs when used with RT is not well established. Multiple case reports and case series have shown a risk of gastrointestinal (GI) toxicity when abdominal sites are treated with radiation, either concurrently with VEGF/VEGFR inhibitors, or as much as 17 months prior to use of these agents [67], [68], [69], [70]. Holding these drugs has an unclear effect on the risk of toxicity, and due to the long half-life of bevacizumab, may not be feasible. The risks and benefits of treatment should be discussed with each individual patient for whom VEGF inhibition and RT is being considered.

BRAF inhibiting agents carry a risk of skin reactions when used concurrently with RT, in addition to other less common toxicities. A literature review and consensus process conducted by the ECOG in 2016 recommended that BRAF inhibitors be held for at least 3 days before and after fractionated RT, and at least 1 day before and after stereotactic radiosurgery (SRS), which we support [71].

On the basis of expert opinion, we recommend holding chemotherapeutic agents when the toxicity of the agent overlaps with the site to be irradiated, such as holding doxorubicin when irradiating a sternal metastasis. We hold these agents for 2 half-lives before and after radiation.

CDK inhibitors have an unclear safety profile when used with radiation. The risk of neutropenia does not appear to be different than with CDK inhibitors alone [72], and a small retrospective study showed combination treatment to be safe [73]. Alternatively, other studies have raised concern for pulmonary, GI, and skin toxicities [74], [75], [76], [77]. Due to the dosing schedule of palbociclib, RT can be delivered during the week when patients are not receiving the drug. For other CDK inhibitors, we recommend holding for 1–2 half-lives before RT and 24 hours after.

Most other chemotherapeutics and targeted agents have not been thoroughly studied in the setting of concurrent use with RT. We generally hold other chemotherapy and targeted agents for 1–2 half-lives before RT and 24 hours after.

## Discussion

This study confirms that a consensus process among multidisciplinary oncologists in an academic-community partnership is feasible, and can address gaps in available national guidelines, even in the absence of level 1 evidence. This process resulted in an updated review of the evidence underpinning local treatment of non-spine bone metastases, including patient selection for surgical management, complex radiation and interventional radiologic approaches, appropriate radiation regimens and field designs, and safe approaches to continuing systemic therapy during radiation.

This initiative provides recommendations for clinicians to use in their practice treating non-spine bone metastases while facilitating opportunities to reduce variation in diverse practice settings which can benefit patients seeking care close to home. Most notably, our recommendation for treatment with 5 or fewer fractions is novel and clinically actionable. It builds upon prior publications advocating for high-value patient-centered short-course regimens [3], [78] by integrating known barriers to uptake of single fraction [54] while allowing newer techniques such as SBRT. It has therefore been selected as the primary endpoint of a future prospective implementation trial.

Our consensus process also elucidated areas with a relative lack of prospective data. Specifically, uncertainty exists about the definition of the oligometastatic state and proper management for these patients. While some patients appear to benefit from metastasis-directed therapy, optimal patient selection remains unclear. Trials are underway, for example, to determine how many metastases can be effectively treated with local therapy [79], the role of RT for asymptomatic lesions in high-risk sites, and the role of pre-operative RT for patients requiring stabilization surgery. Other areas that require further study are pathologic fracture prediction models, and the optimal candidates for percutaneous interventions. Nonetheless, a framework for considering these emerging techniques is provided.

Our study does have several limitations. First, both topic questions and responses may be biased by the experts selected for the panel. However, we conducted a systematic literature review and included multi-disciplinary physicians from both academic and community-based practice to represent a range of perspectives with the intent of being generalizable. Second, our consensus process was conducted within the context of an existing academic-community partnership, and therefore certain recommendations may be difficult to implement in other settings. Nonetheless, these recommendations may still help oncologists determine when referral is potentially beneficial. Third, several of the questions that were identified for consensus lacked high-level evidence, given the rapidly changing nature of the field. We chose to provide recommendations based upon the available data and expert opinion on such topics, in response to a survey of oncologists showing that in the absence of high-level evidence, clinicians prefer guidelines based upon expert opinion to no guidelines [80].

Our results are relevant to practicing oncologists treating non-spine bone metastases, as well as clinical researchers, highlighting areas where further study is needed. As treatment for metastatic cancer continues to evolve, it is crucial that efforts are made to ensure the benefits of treatment are provided to all patients, regardless of practice setting and geographic location.

## Declaration of Competing Interest

The authors declare that they have no known competing financial interests or personal relationships that could have appeared to influence the work reported in this paper.

## References

[1] Hernandez R.K., Wade S.W., Reich A., Pirolli M., Liede A., Lyman G.H. (2018). Incidence of bone metastases in patients with solid tumors: analysis of oncology electronic medical records in the United States. BMC Cancer.

[2] Cox B.W., Spratt D.E., Lovelock M., Bilsky M.H., Lis E., Ryu S. (2012). International Spine Radiosurgery Consortium consensus guidelines for target volume definition in spinal stereotactic radiosurgery. Int J Radiat Oncol Biol Phys.

[3] Lutz S., Balboni T., Jones J., Lo S., Petit J., Rich S.E. (2017). Palliative radiation therapy for bone metastases: update of an ASTRO Evidence-Based Guideline. Pract Radiat Oncol.

[4] Grávalos C., Rodríguez C., Sabino A., Seguí M.Á., Virizuela J.A., Carmona A. (2016). SEOM clinical guideline for bone metastases from solid tumours (2016). Clin Transl Oncol.

[5] Gillespie E.F., Lapen K., Wang D.G. (2020). Replacing 30 Gy in 10 fractions with stereotactic body radiation therapy for bone metastases: a large multi-site single institution experience 2016–2018. Clin Transl Radiat Oncol.

[6] Santos P.M.G., Lapen K., Zhang Z. (2020). Trends in radiotherapy for bone metastases, 2015–2017: choosing Wisely in the era of complex radiation. Int J Radiat Oncol Biol Phys.

[7] Radiation Reimagined: How MSK Experts in Radiation Oncology Are Transforming Care for Metastatic Cancer, MSK News, Memorial Sloan Kettering Cancer Center, 2021.

[8] Eubank B.H., Mohtadi N.G., Lafave M.R., Wiley J.P., Bois A.J., Boorman R.S. (2016). Using the modified Delphi method to establish clinical consensus for the diagnosis and treatment of patients with rotator cuff pathology. BMC Med Res Methodol.

[9] Dalkey NC: The Delphi Method: An Experimental Study of Group Opinion, in Corporation TR (ed), 1969.

[10] Gustafson D.H., Shukla R.K., Delbecq A., Walster G.W. (1973). A comparative study of differences in subjective likelihood estimates made by individuals, interacting groups, Delphi groups, and nominal groups. Organiz Behav Hum Perform.

[11] Turner G.H., Weiner D.K. (2002). Essential components of a medical student curriculum on chronic pain management in older adults: results of a modified delphi process. Pain Med.

[12] Ebell M.H., Siwek J., Weiss B.D. (2004). Strength of recommendation taxonomy (SORT): a patient-centered approach to grading evidence in the medical literature. Am Fam Physician.

[13] Nguyen T.K., Sahgal A., Dagan R., Eppinga W., Guckenberger M., Kim J.H. (2020). Stereotactic body radiation therapy for nonspine bone metastases: international practice patterns to guide treatment planning. Pract Radiat Oncol.

[14] Weeks J.C., Cook E.F., O'Day S.J. (1998). Relationship between cancer patients' predictions of prognosis and their treatment preferences. JAMA.

[15] Matsuyama R., Reddy S., Smith T.J. (2006). Why do patients choose chemotherapy near the end of life? A review of the perspective of those facing death from cancer. J Clin Oncol.

[16] Wolfe J., Klar N., Grier H.E., Duncan J., Salem-Schatz S., Emanuel E.J. (2000). Understanding of prognosis among parents of children who died of cancer: impact on treatment goals and integration of palliative care. JAMA.

[17] Weeks J.C., Catalano P.J., Cronin A., Finkelman M.D., Mack J.W., Keating N.L. (2012). Patients’ expectations about effects of chemotherapy for advanced cancer. N Engl J Med.

[18] Mack J.W., Weeks J.C., Wright A.A., Block S.D., Prigerson H.G. (2010). End-of-life discussions, goal attainment, and distress at the end of life: predictors and outcomes of receipt of care consistent with preferences. J Clin Oncol.

[19] Sapir R., Catane R., Kaufman B., Isacson R., Segal A., Wein S. (2000). Cancer patient expectations of and communication with oncologists and oncology nurses: the experience of an integrated oncology and palliative care service. Support Care Cancer.

[20] Gripp S., Moeller S., Bölke E., Schmitt G., Matuschek C., Asgari S. (2007). Survival prediction in terminally ill cancer patients by clinical estimates, laboratory tests, and self-rated anxiety and depression. J Clin Oncol.

[21] Maltoni M., Pirovano M., Scarpi E. (1995). Prediction of survival of patients terminally ill with cancer. Results of an Italian prospective multicentric study. Cancer.

[22] Reuben D.B., Mor V., Hiris J. (1988). Clinical symptoms and length of survival in patients with terminal cancer. Arch Intern Med.

[23] Allard P., Dionne A., Potvin D. (1995). Factors associated with length of survival among 1081 terminally ill cancer patients. J Palliat Care.

[24] Rosenthal M.A., Gebski V.J., Kefford R.F., Stuart-Harris R.C. (1993). Prediction of life-expectancy in hospice patients: identification of novel prognostic factors. Palliat Med.

[25] Evans C., Mccarthy M. (1985). Prognostic uncertainty in terminal care: can the Karnofsky index help?. Lancet.

[26] Maltoni M., Nanni O., Derni S., Innocenti M.P., Fabbri L., Riva N. (1994). Clinical prediction of survival is more accurate than the Karnofsky performance status in estimating life span of terminally ill cancer patients. Eur J Cancer.

[27] Albain K.S., Crowley J.J., LeBlanc M., Livingston R.B. (1991). Survival determinants in extensive-stage non-small-cell lung cancer: the Southwest Oncology Group experience. J Clin Oncol.

[28] Cheon P.M., Wong E., Thavarajah N., Dennis K., Lutz S., Zeng L. (2015). A definition of “uncomplicated bone metastases” based on previous bone metastases radiation trials comparing single-fraction and multi-fraction radiation therapy. J Bone Oncol.

[29] Rich S.E., Chow R., Raman S., Liang Zeng K., Lutz S., Lam H. (2018). Update of the systematic review of palliative radiation therapy fractionation for bone metastases. Radiother Oncol.

[30] Chow R., Hoskin P., Schild S.E., Raman S., Im J., Zhang D. (2019). Single vs multiple fraction palliative radiation therapy for bone metastases: cumulative meta-analysis. Radiother Oncol.

[31] Fischberg D., Bull J., Casarett D. (2013). Five things physicians and patients should question in hospice and palliative medicine. J Pain Symptom Manage.

[32] Philipp T.C., Mikula J.D., Doung Y.C. (2020). Is there an association between prophylactic femur stabilization and survival in patients with metastatic bone disease?. Clin Orthop Relat Res.

[33] Forsberg J.A., Wedin R., Boland P.J. (2017). Can we estimate short- and intermediate-term survival in patients undergoing surgery for metastatic bone disease?. Clin Orthop Relat Res.

[34] Investigators H., Bhandari M., Einhorn T.A. (2019). Total hip arthroplasty or hemiarthroplasty for hip fracture. N Engl J Med.

[35] Damron T.A., Nazarian A., Entezari V. (2016). CT-based structural rigidity analysis is more accurate than mirels scoring for fracture prediction in metastatic femoral lesions. Clin Orthop Relat Res.

[36] Scott E, Klement MR, Brigman BE, et al: Beyond Mirels: Factors Influencing Surgical Outcome of Metastasis to the Extremities in the Modern Era. J Surg Orthop Adv 27:178-186. 30489242

[37] Siegel G.W., Biermann J.S., Calinescu A.A. (2018). Surgical approach to bone metastases. Curr Osteoporos Rep.

[38] Tatar Z., Soubrier M., Dillies A.F. (2014). Assessment of the risk factors for impending fractures following radiotherapy for long bone metastases using CT scan-based virtual simulation: a retrospective study. Radiat Oncol.

[39] Mirels H. (1989). Metastatic disease in long bones. A proposed scoring system for diagnosing impending pathologic fractures. Clin Orthop Relat Res.

[40] Coupal T.M., Pennycooke K., Mallinson P.I. (2017). The hopeless case? Palliative cryoablation and cementoplasty procedures for palliation of large pelvic bone metastases. Pain Physician.

[41] Moser T.P., Onate M., Achour K. (2019). Cementoplasty of pelvic bone metastases: systematic assessment of lesion filling and other factors that could affect the clinical outcomes. Skeletal Radiol.

[42] Anselmetti G.C., Manca A., Ortega C. (2008). Treatment of extraspinal painful bone metastases with percutaneous cementoplasty: a prospective study of 50 patients. Cardiovasc Intervent Radiol.

[43] Sun Y., Zhang H., Xu H.R. (2019). Analgesia of percutaneous thermal ablation plus cementoplasty for cancer bone metastases. J Bone Oncol.

[44] Cazzato R.L., Palussiere J., Buy X. (2015). Percutaneous long bone cementoplasty for palliation of malignant lesions of the limbs: a systematic review. Cardiovasc Intervent Radiol.

[45] Garnon J., Meylheuc L., Cazzato R.L. (2019). Percutaneous extra-spinal cementoplasty in patients with cancer: a systematic review of procedural details and clinical outcomes. Diagn Interv Imaging.

[46] Chow E., Meyer R.M., Chen B.E. (2014). Impact of reirradiation of painful osseous metastases on quality of life and function: a secondary analysis of the NCIC CTG SC.20 randomized trial. J Clin Oncol.

[47] Chow E., van der Linden Y.M., Roos D. (2014). Single versus multiple fractions of repeat radiation for painful bone metastases: a randomised, controlled, non-inferiority trial. Lancet Oncol.

[48] Goetz M.P., Callstrom M.R., Charboneau J.W. (2004). Percutaneous image-guided radiofrequency ablation of painful metastases involving bone: a multicenter study. J Clin Oncol.

[49] van der Linden Y.M., Lok J.J., Steenland E. (2004). Single fraction radiotherapy is efficacious: a further analysis of the Dutch Bone Metastasis Study controlling for the influence of retreatment. Int J Radiat Oncol Biol Phys.

[50] Roos D.E., Turner S.L., O'Brien P.C. (2005). Randomized trial of 8 Gy in 1 versus 20 Gy in 5 fractions of radiotherapy for neuropathic pain due to bone metastases (Trans-Tasman Radiation Oncology Group, TROG 96.05). Radiother Oncol.

[51] 8 Gy single fraction radiotherapy for the treatment of metastatic skeletal pain: randomised comparison with a multifraction schedule over 12 months of patient follow-up. Bone Pain Trial Working Party. Radiother Oncol 52:111-21, 1999. 10577696

[52] Cole D.J. (1989). A randomized trial of a single treatment versus conventional fractionation in the palliative radiotherapy of painful bone metastases. Clin Oncol (R Coll Radiol).

[53] Hartsell W.F., Scott C.B., Bruner D.W. (2005). Randomized trial of short- versus long-course radiotherapy for palliation of painful bone metastases. J Natl Cancer Inst.

[54] Squires J.E., Asad S., Varin M.D. (2021). Behavioral determinants of Canadian radiation oncologists; use of single fraction palliative radiation therapy for uncomplicated bone metastases. Int J Radiat Oncol Biol Phys.

[55] El Hawwari B.T. (2012). A.: Comparison of 8Gy single fraction radiotherapy versus 20Gy in five fractions Or 30Gy in 10 fractions for the treatment of metastatic bone pain. Ann Oncol.

[56] Sprave T., Verma V., Forster R. (2018). Randomized phase II trial evaluating pain response in patients with spinal metastases following stereotactic body radiotherapy versus three-dimensional conformal radiotherapy. Radiother Oncol.

[57] Nguyen Q.N., Chun S.G., Chow E. (2019). Single-fraction stereotactic vs conventional multifraction radiotherapy for pain relief in patients with predominantly nonspine bone metastases: a randomized phase 2 trial. JAMA Oncol.

[58] Sahgal A.M.S., Siva S., Masucci L., Foote M.C., Brundage M., Butler J. (2020). LBA 2 CCTG SC.24/TROG 17.06: a randomized phase II/III study Comparing 24Gy in 2 stereotactic body radiotherapy (SBRT) fractions versus 20Gy in 5 Conventional Palliative Radiotherapy (CRT) fractions for patients with painful spinal metastases. Am Soc Radiat Oncol.

[59] Gomez D.R., Tang C., Zhang J. (2019). Local Consolidative therapy Vs. maintenance therapy or observation for patients with oligometastatic non-small-cell lung cancer: long-term results of a multi-institutional, Phase II, randomized study. J Clin Oncol.

[60] Palma D.A., Olson R., Harrow S. (2019). Stereotactic ablative radiotherapy versus standard of care palliative treatment in patients with oligometastatic cancers (SABR-COMET): a randomised, phase 2, open-label trial. Lancet.

[61] Epstein-Peterson Z.D., Sullivan A., Krishnan M. (2015). Postoperative radiation therapy for osseous metastasis: outcomes and predictors of local failure. Pract Radiat Oncol.

[62] Elhammali A., Milgrom S.A., Amini B. (2019). Postoperative radiotherapy for multiple myeloma of long bones: should the entire rod be treated?. Clin Lymphoma Myeloma Leuk.

[63] Hiniker S.M., Reddy S.A., Maecker H.T. (2016). A prospective clinical trial combining radiation therapy with systemic immunotherapy in metastatic melanoma. Int J Radiat Oncol Biol Phys.

[64] Welsh J.W., Heymach J.V., Chen D. (2020). Phase I trial of pembrolizumab and radiation therapy after induction chemotherapy for extensive-stage small cell lung cancer. J Thorac Oncol.

[65] Ahn P.H., Vu H.T., Lannin D. (2005). Sequence of radiotherapy with tamoxifen in conservatively managed breast cancer does not affect local relapse rates. J Clin Oncol.

[66] Pierce L.J., Hutchins L.F., Green S.R. (2005). Sequencing of tamoxifen and radiotherapy after breast-conserving surgery in early-stage breast cancer. J Clin Oncol.

[67] Lordick F., Geinitz H., Theisen J. (2006). Increased risk of ischemic bowel complications during treatment with bevacizumab after pelvic irradiation: report of three cases. Int J Radiat Oncol Biol Phys.

[68] Barney B.M., Markovic S.N., Laack N.N. (2013). Increased bowel toxicity in patients treated with a vascular endothelial growth factor inhibitor (VEGFI) after stereotactic body radiation therapy (SBRT). Int J Radiat Oncol Biol Phys.

[69] Brade A.M., Ng S., Brierley J. (2016). Phase 1 trial of sorafenib and stereotactic body radiation therapy for hepatocellular carcinoma. Int J Radiat Oncol Biol Phys.

[70] Pollom E.L., Deng L., Pai R.K. (2015). Gastrointestinal toxicities with combined antiangiogenic and stereotactic body radiation therapy. Int J Radiat Oncol Biol Phys.

[71] Anker C.J., Grossmann K.F., Atkins M.B. (2016). Avoiding severe toxicity from combined BRAF inhibitor and radiation treatment: consensus guidelines from the eastern cooperative oncology group (ECOG). Int J Radiat Oncol Biol Phys.

[72] Guerini A.E., Pedretti S., Salah E. (2020). A single-center retrospective safety analysis of cyclin-dependent kinase 4/6 inhibitors concurrent with radiation therapy in metastatic breast cancer patients. Sci Rep.

[73] Beddok A., Xu H.P., Henry A.A. (2020). Concurrent use of palbociclib and radiation therapy: single-centre experience and review of the literature. Br J Cancer.

[74] David S., Ho G., Day D. (2021). Enhanced toxicity with CDK 4/6 inhibitors and palliative radiotherapy: non-consecutive case series and review of the literature. Transl Oncol.

[75] Messer J.A., Ekinci E., Patel T.A. (2019). Enhanced dermatologic toxicity following concurrent treatment with palbociclib and radiation therapy: a case report. Rep Pract Oncol Radiother.

[76] Kawamoto T., Shikama N., Sasai K. (2019). Severe acute radiation-induced enterocolitis after combined palbociclib and palliative radiotherapy treatment. Radiother Oncol.

[77] Bosacki C., Bouleftour W., Sotton S. (2021). CDK 4/6 inhibitors combined with radiotherapy: a review of literature. Clin Transl Radiat Oncol.

[78] Hahn C., Kavanagh B., Bhatnagar A. (2014). Choosing wisely: the American Society for Radiation Oncology’s top 5 list. Pract Radiat Oncol.

[79] Palma D.A., Olson R., Harrow S. (2019). Stereotactic ablative radiotherapy for the comprehensive treatment of 4–10 oligometastatic tumors (SABR-COMET-10): study protocol for a randomized phase III trial. BMC Cancer.

[80] Dillmon M, Goldberg JM, Ramalingam SS, et al: Clinical practice guidelines for cancer care: utilization and expectations of the practicing oncologist. J Oncol Pract 8:350-3, 2 p following 353, 2012. 10.1200/JOP.2012.000599 PMC3500479 23598844

