# Supplementary material for: Multidisciplinary Treatment of Non-Spine Bone Metastases: Results of a Modified Delphi Consensus Process
Source: Clin Transl Radiat Oncol. 2022 Apr 26;35:76–83. doi: 10.1016/j.ctro.2022.04.009 (PMC9127274; doi:10.1016/j.ctro.2022.04.009)
Supplement: Supplementary data 1 [file mmc1.zip › Supplemental File 1_Survey of clinical questions.pdf]

MSK Alliance - Topics for consensus

Each question below represents a potential topic for consensus in management of NON-SPINE BONE METASTASES.

Please RATE THE IMPORTANCE of each topic, to allow us to prioritize as we develop standards of care for MSKCC and Alliance sites.

* Required

DOSE AND FRACTIONATION (4 QUESTIONS)

1. What fractionation schemes are MOST EFFECTIVE for the treatment of pain and/or prevention of morbidity from non-spine bone metastases?

Mark only one oval.

	1	2	3	4	5	
Not important	◯	◯	◯	◯	◯	Essential

2. What fractionation schemes are MOST EFFECTIVE for ablative radiation in the setting of oligometastatic disease?

Mark only one oval.

	1	2	3	4	5	
Not important	◯	◯	◯	◯	◯	Essential

3. When is SBRT PREFERRED for the treatment of non-spinal bone metastases?

Mark only one oval.

	1	2	3	4	5	
Not important	◯	◯	◯	◯	◯	Essential

4. When is 8Gyx1 PREFERRED for the treatment of pain and/or prevention of morbidity from non-spine bone metastases?

Mark only one oval.

	1	2	3	4	5	
Not important	◯	◯	◯	◯	◯	Essential

CLINICAL TOOLS FOR ROUTINE PRACTICE (2 QUESTIONS)

5. What tools should be used to estimate performance status (and/or prognosis) in the setting of metastatic disease?

Mark only one oval.

	1	2	3	4	5	
Not important	◯	◯	◯	◯	◯	Essential

6. What patient-reported outcomes (PROs) should be collected in routine practice for patients undergoing local therapy for non-spine bone metastases?

Mark only one oval.

	1	2	3	4	5	
Not important	⬭	⬭	⬭	⬭	⬭	Essential

TREATMENT PLANNING (3 QUESTIONS)

7. What are the desired margins and elective volumes for SBRT in cases of non-spinal bone mets? (Will include a consensus contouring exercise with 5 example cases)

Mark only one oval.

	1	2	3	4	5	
Not important	⬭	⬭	⬭	⬭	⬭	Essential

8. When should an MRI be used for treatment planning?

Mark only one oval.

	1	2	3	4	5	
Not important	⬭	⬭	⬭	⬭	⬭	Essential

9. What normal organs (OARs) should be contoured for tumors in a) pelvis, b) hip, c) shoulder?

Mark only one oval.

	1	2	3	4	5	
Not important	◯	◯	◯	◯	◯	Essential

COMPLICATED CASES (2 QUESTIONS)

10. What clinical scenarios can be classified as “complex” and warrant review by a multidisciplinary team?

Mark only one oval.

	1	2	3	4	5	
Not important	◯	◯	◯	◯	◯	Essential

11. What are important considerations for re-irradiation of non-spine bone mets? (i.e. composite dose, dose constraints, dose de-escalation)

Mark only one oval.

	1	2	3	4	5	
Not important	◯	◯	◯	◯	◯	Essential

MULTIDISCIPLINARY TOPICS (6 QUESTIONS)

12. When should a patient with non-spine bone mets be referred to radiation oncology for evaluation? (pain, oligomet, high-risk of fracture, etc)

Mark only one oval.

	1	2	3	4	5	
Not important	◯	◯	◯	◯	◯	Essential

13. When should a patient be referred for surgical fixation/stabilization surgery?

Mark only one oval.

	1	2	3	4	5	
Not important	◯	◯	◯	◯	◯	Essential

14. What is the preferred approach to radiotherapy in the setting of stabilization surgery?

Mark only one oval.

	1	2	3	4	5	
Not important	◯	◯	◯	◯	◯	Essential

15. When should image-guided ablation (or other percutaneous techniques like cementoplasty) be considered for non-spine bone metastases?

Mark only one oval.

	1	2	3	4	5	
Not important	◯	◯	◯	◯	◯	Essential

16. What role do bone-modifying agents (bisphosphonates, denosumab) play in the management of non-spine bone metastases?

Mark only one oval.

	1	2	3	4	5	
Not important	◯	◯	◯	◯	◯	Essential

17. What systemic therapies should be held during radiation for non-spine bone metastases and for how long?

Mark only one oval.

	1	2	3	4	5	
Not important	◯	◯	◯	◯	◯	Essential

18. Your name *

____

19. Other ideas/comments:

____
____
____
____
____

This content is neither created nor endorsed by Google.

Google Forms
