# Supplementary material for: Multidisciplinary Treatment of Non-Spine Bone Metastases: Results of a Modified Delphi Consensus Process
Source: Clin Transl Radiat Oncol. 2022 Apr 26;35:76–83. doi: 10.1016/j.ctro.2022.04.009 (PMC9127274; doi:10.1016/j.ctro.2022.04.009)
Supplement: Supplementary data 2 [file mmc2.zip › Supplemental File 2_Q selection.docx]

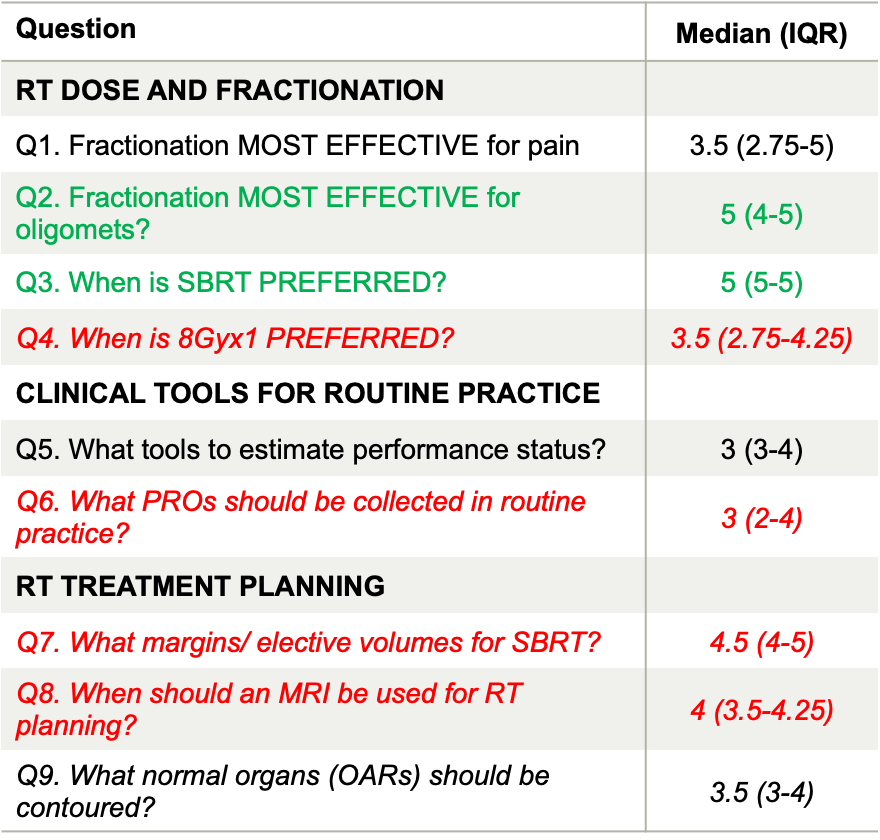

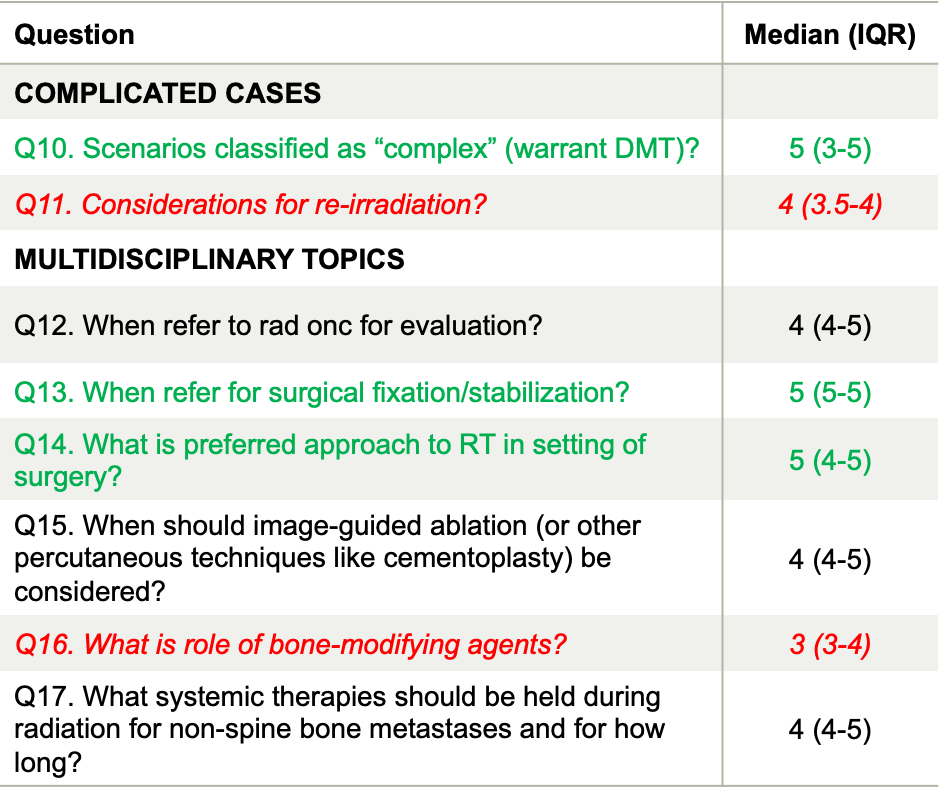
**
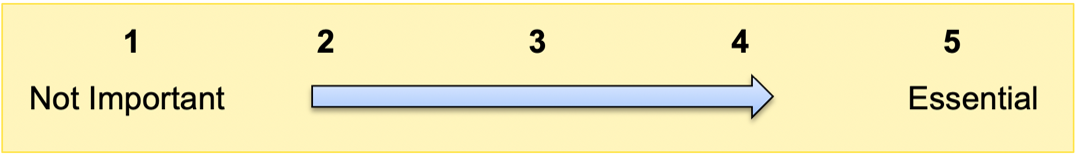
Supplement Table 1.** Survey results of clinical question importance ratings were presented to the initial expert panel for selection. Eleven questions advanced to the next phase including all with a median score of 5 (green). Six questions did not advance to the next phase (red).
