# Supplementary material for: Multidisciplinary Treatment of Non-Spine Bone Metastases: Results of a Modified Delphi Consensus Process
Source: Clin Transl Radiat Oncol. 2022 Apr 26;35:76–83. doi: 10.1016/j.ctro.2022.04.009 (PMC9127274; doi:10.1016/j.ctro.2022.04.009)

**Supplemental File 1.** Example of consensus question and answer statement development process including evidence informing recommendations and iterative revisions.

**MSK Alliance/NCCN Non-Spine Bone Mets Consensus Project**

***Thank you*** *for championing the following question(s)! For questions or concerns, please contact Co-PI Dr. Erin Gillespie (*[*gillespe@mskcc.org*](mailto:gillespe@mskcc.org)*) or med student research assistant Kaitlyn Lapen (*[*lapenk@mskcc.org*](mailto:lapenk@mskcc.org)*).*

**INSTRUCTIONS:**

1. Review/update the list of papers below that will provide the evidence for your statement.
   1. Cross out/delete any that are not relevant.
   2. Add the title and/or PMIDs for any papers you would like to add.
2. Draft an Answer statement that summarizes the essence of the papers.
   1. NOTE: This statement will be reviewed/circulated to the larger group of 12 bone mets experts.
3. (**Recommended**) Assign a level of evidence for your statement
4. *(Optional)* If you would like to modify the wording of the Question, or break into 2 questions, that is totally fine! Just please use track changes.

**YOUR QUESTION:**

##### Q13. When should a patient with non-spine bone metastases be referred for prophylactic surgical fixation/stabilization?

**ANSWER (Initial draft):**

**Q13 Answer *(Initial Draft by orthopaedic surgery experts)*.** Readily available morphology-based scoring tools for predicting pathologic long bone fracture have limited accuracy. Prediction algorithms with higher accuracy require specialized or proprietary instruments and are not in widespread clinical use. In recognition of the significant morbidity associated with pathologic long bone fractures, the increased mortality associated with pathologic femur fractures, and the improved outcomes associated with stabilization of impending (rather than completed) femur fracture, clinicians should aim to promptly refer for surgical evaluation those patients at imminent risk of pathologic long bone fracture, particularly in the femur. Patients should be referred if their medical status and oncologic life expectancy are permissive of surgery and any of the following are present: lytic long bone lesion with pain that is worsened with activity, any significant lesion in the femur that is either lytic or painful, progressive growth after radiation, or failure of palliation with radiation.

**Q13 Answer *(Final Draft after Steering Committee revision and 2 Rounds of consensus)***

Referral to a surgeon should be considered if the patient’s medical status and oncologic life expectancy are permissive of surgery and any of the following are present:

1. Lytic long bone or pelvic lesion with pain that is worsened with activity,
2. Any significant lesion in the femur that is either lytic or painful,
3. Progressive growth after radiation, or
4. Failure of palliation with radiation

**EXAMPLE:**


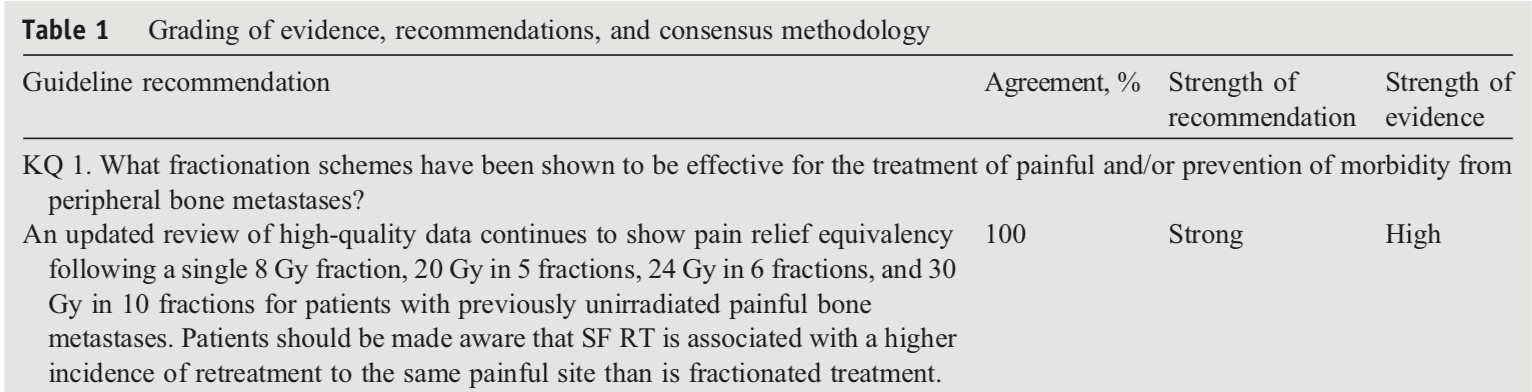


**EVIDENCE TO SUPPORT YOUR ANSWER:**

**Q13.** When should a patient with non-spine bone metastases be referred for surgical fixation/stabilization?

**Search**

((“bone metastasis” OR “bone metastases”) AND (“Surgical Procedures, Operative”[Mesh] OR “surgery” OR “fixation” OR “stabilization”) AND ("trial" or "randomized"))

Filters: Publication date from 2010/01/01 to 2020/03/23, Humans, English, Adult: 19+ years

Results: 142 🡪 The following publications were selected from this list based on title/abstract review.

| **Articles** | | | | |
| --- | --- | --- | --- | --- |
| Title | First Author | Year | Pubmed Link | Study Design/Notes |
| Metastatic Disease in Long Bones. A Proposed Scoring System for Diagnosing Impending Pathologic Fractures | Mirels | 1989 | [2684463](https://pubmed.ncbi.nlm.nih.gov/2684463/) | Retrospective review |
| CT-based Structural Rigidity Analysis Is More Accurate Than Mirels Scoring for Fracture Prediction in Metastatic Femoral Lesions | Damron | 2016 | [26169800](https://www.ncbi.nlm.nih.gov/pmc/articles/PMC4746194/) | Prospective observational study |
| Surgical Approach to Bone Metastases | Siegel | 2018 | [29915966](https://pubmed.ncbi.nlm.nih.gov/29915966/) | Systematic review |
| Beyond Mirels: Factors Influencing Surgical Outcome of Metastasis to the Extremities in the Modern Era | Scott | 2018 | [30489242](https://pubmed.ncbi.nlm.nih.gov/30489242/) | Retrospective review |
| Treatment Planning and Fracture Prediction in Patients with Skeletal Metastasis with CT-Based Rigidity Analysis. | Nazarian | 2015 | [25724521](https://www.ncbi.nlm.nih.gov/pubmed/25724521) | Prospective observational study |
| Surgical treatment for periacetabular metastatic lesions. | Charles | 2017 | [28483275](https://www.ncbi.nlm.nih.gov/pubmed/28483275) | Retrospective review |
| Surgical Treatment in Bone Metastases in the Appendicular Skeleton | Clara-  Altamirano | 2018 | [29574162](https://pubmed.ncbi.nlm.nih.gov/29574162/) | Retrospective review |
| Analysis of orthopedic surgery of bone metastases in breast cancer patients. | Wegener | 2012 | [23181392](https://www.ncbi.nlm.nih.gov/pubmed/23181392) | Retrospective review |
| Assessment of the risk factors for impending fractures following radiotherapy for long bone metastases using CT scan-based virtual simulation: a retrospective study. | Tatar | 2014 | [25319635](https://www.ncbi.nlm.nih.gov/pubmed/25319635) | Retrospective review |
| Radiological response and clinical outcome in patients with femoral bone metastases after radiotherapy. | Harada | 2010 | [19934590](https://www.ncbi.nlm.nih.gov/pubmed/19934590) | Retrospective review |
| Multimodal Treatment of Bone Metastasis—A Surgical Perspective | Soeharno | 2018 | [30245668](https://www.ncbi.nlm.nih.gov/pubmed/30245668) | Review |
| Simple Radiographic Parameter Predicts Fracturing in Metastatic femoral Bone Lesions: Results From a Randomised Trial | Van der Linden | 2003 | [14597353](https://pubmed.ncbi.nlm.nih.gov/14597353/) | Randomized Trial |
| FDG PET/CT Assesses the Risk of Femoral Pathological Fractures in Patients With Metastatic Breast Cancer | Ulaner | 2017 | [28166159](https://www.ncbi.nlm.nih.gov/pmc/articles/PMC5334437/) | Retrospective review |
| Can We Estimate Short- And Intermediate-term Survival in Patients Undergoing Surgery for Metastatic Bone Disease? | Forsberg | 2017 | [27909972](https://www.ncbi.nlm.nih.gov/pmc/articles/PMC5339146/) | Retrospective review with external validation |
| Is There an Association Between Prophylactic Femur Stabilization and Survival in Patients With Metastatic Bone Disease? | Phillip | 2020 | [32168065](https://pubmed.ncbi.nlm.nih.gov/32168065/) | Retrospective review |
| Patient Survival After Hip Arthroplasty for Metastatic Disease of the Hip | Schneiderbauer | 2004 | [15292415](https://pubmed.ncbi.nlm.nih.gov/15292415/) | Retrospective review |

##### LEVEL OF EVIDENCE: Low to Moderate

##### Please state the approximate “Level of Evidence” of data supporting your statement based on the following scoring system provided by US Preventive Services Task Force ([Link to information](https://www.uspreventiveservicestaskforce.org/uspstf/us-preventive-services-task-force-ratings)):


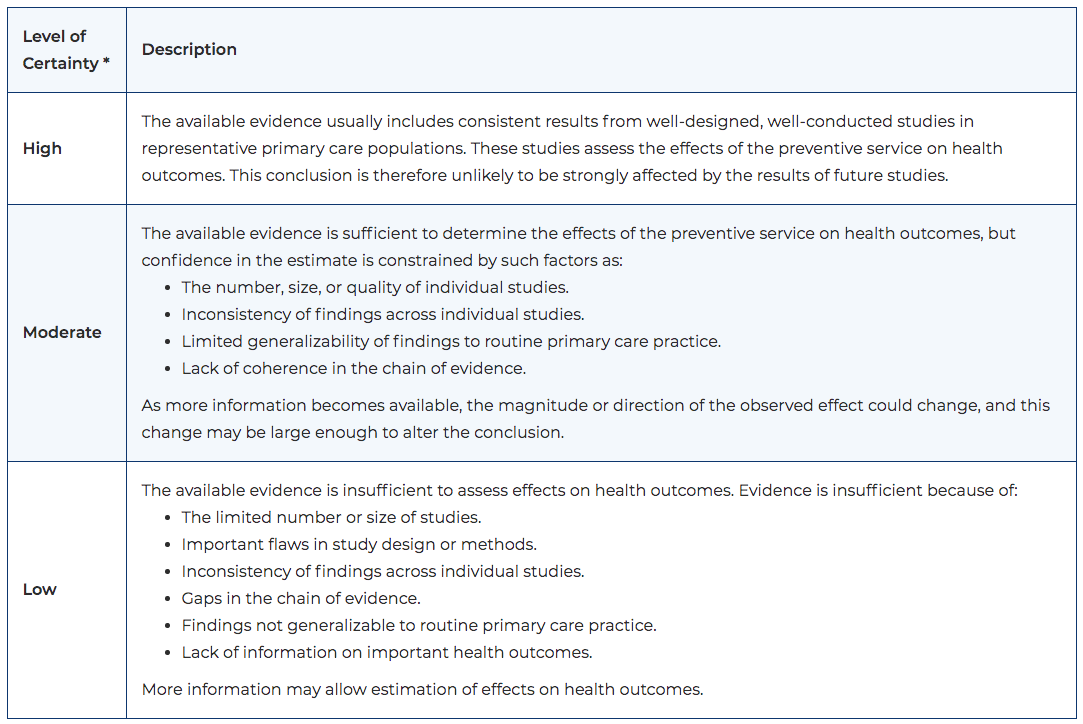

Supplement: Supplementary data 3 [file mmc3.zip › Supplemental File 3_Q13 example.docx]
