# Supplementary material for: Multidisciplinary Treatment of Non-Spine Bone Metastases: Results of a Modified Delphi Consensus Process
Source: Clin Transl Radiat Oncol. 2022 Apr 26;35:76–83. doi: 10.1016/j.ctro.2022.04.009 (PMC9127274; doi:10.1016/j.ctro.2022.04.009)
Supplement: Supplementary data 4 [file mmc4.zip › Supplemental Materials 2.docx]

**Supplemental Materials 2:** MeSH search terms used in the literature review provided to the initial expert panel to guide drafting of question/answer statements.

What fractionation schemes are MOST EFFECTIVE for the treatment of pain and/or prevention of morbidity from non-spinal bone metastases?

((“bone metastasis” OR “bone metastases”) AND (“Radiotherapy”[Mesh] OR “Radiation”[Mesh] OR “Radiotherapy, Conformal”[Mesh] OR “Radiosurgery”[Mesh] OR “Radiotherapy, Intensity-Modulated”[Mesh] OR “radiotherapy” OR “radiation”) AND (“fractionation” OR “fraction” OR “dose”) AND ("trial" OR "randomized"))

Filters: Publication date from 2010/01/01 to 2020/03/23, Humans, English, Adult: 19+ years

What fractionation schemes are MOST EFFECTIVE for ablative radiation in the setting of oligometastatic disease?

((“bone metastasis” OR “oligometastases” OR “oligometastatic”) AND (“Radiotherapy”[Mesh] OR “Radiation”[Mesh] OR “Radiotherapy, Conformal”[Mesh] OR “Radiosurgery”[Mesh] OR “Radiotherapy, Intensity-Modulated”[Mesh] OR “radiotherapy” OR “radiation”) AND (“stereotactic body” OR “stereotactic” OR “ablative” OR “ablation”) AND ("trial" OR "randomized"))

Filters: Publication date from 2010/01/01 to 2020/04/14, Humans, English, Adult: 19+ years

When is SBRT preferred for the treatment of non-spinal bone metastases? (i.e. palliation of pain, oligomets, other?)

((“bone metastasis” OR “bone metastases”) AND (“Radiotherapy”[Mesh] OR “Radiation”[Mesh] OR “Radiotherapy, Conformal”[Mesh] OR “Radiosurgery”[Mesh] OR “Radiotherapy, Intensity-Modulated”[Mesh] OR “radiotherapy” OR “radiation”) AND (“stereotactic body” OR “stereotactic”))

Filters: Publication date from 2010/01/01 to 2020/03/23, Humans, English, Adult: 19+ years

When is 8Gyx1 preferred for the treatment of pain and/or prevention of morbidity from non-spinal bone metastases?

((“bone metastasis” OR “bone metastases”) AND (“Radiotherapy”[Mesh] OR “Radiation”[Mesh] OR “Radiotherapy, Conformal”[Mesh] OR “Radiosurgery”[Mesh] OR “Radiotherapy, Intensity-Modulated”[Mesh] OR “radiotherapy” OR “radiation”) AND (“fractionation” OR “fraction” OR “dose” OR “single” OR “hypofractionated”) AND ("trial" OR "randomized"))

Filters: Publication date from 2010/01/01 to 2020/03/23, Humans, English, Adult: 19+ years

What tools should be used to estimate performance status (and/or prognosis) in the setting of metastatic disease?

((“bone metastasis” OR “bone metastases”) AND (“Radiotherapy”[Mesh] OR “Radiation”[Mesh] OR “Radiotherapy, Conformal”[Mesh] OR “Radiosurgery”[Mesh] OR “Radiotherapy, Intensity-Modulated”[Mesh] OR “radiotherapy” OR “radiation”) AND (“Prognosis”[Mesh] OR “functional status” OR “performance status” OR “Health Status Indicators” [Mesh]))

Filters: Publication date from 2010/01/01 to 2020/03/17, Humans, English, Adult: 19+ years

What patient-reported outcomes (PROs) should be collected in routine practice for patients undergoing local therapy for non-spinal bone metastases?

((“bone metastasis” OR “bone metastases”) AND (“Radiotherapy”[Mesh] OR “Radiation”[Mesh] OR “Radiotherapy, Conformal”[Mesh] OR “Radiosurgery”[Mesh] OR “Radiotherapy, Intensity-Modulated”[Mesh] OR “radiotherapy” OR “radiation”) AND (“patient-reported” OR “patient reported” OR “patient-reported outcomes” OR “patient reported outcomes”))

Filters: Publication date from 2010/01/01 to 2020/03/23, Humans, English, Adult: 19+ years

What are the desired margins and elective volumes for SBRT in cases of non-spinal bone mets?

((“bone metastasis” OR “bone metastases”) AND (“Radiotherapy”[Mesh] OR “Radiation”[Mesh] OR “Radiotherapy, Conformal”[Mesh] OR “Radiosurgery”[Mesh] OR “Radiotherapy, Intensity-Modulated”[Mesh] OR “radiotherapy” OR “radiation”) AND (“contours” OR “delineation” OR “margins” OR “volume” OR “contouring”) AND (“stereotactic” OR “stereotactic body”))

Filters: Publication date from 2010/01/01 to 2020/03/23, Humans, English, Adult: 19+ years

When should an MRI be used for treatment planning?

((“bone metastasis” OR “bone metastases”) AND (“Radiotherapy”[Mesh] OR “Radiation”[Mesh] OR “Radiotherapy, Conformal”[Mesh] OR “Radiosurgery”[Mesh] OR “Radiotherapy, Intensity-Modulated”[Mesh] OR “radiotherapy” OR “radiation”) AND (“contours” OR “delineation” OR “margins” OR “volume” OR “contouring” OR “treatment planning”) AND (“magnetic resonance imaging” OR “MRI”))

Filters: Publication date from 2010/01/01 to 2020/03/23, Humans, English, Adult: 19+ years

What normal organs (OARs) should be contoured for tumors in a) hip, b) pelvis, c) shoulder?

((“bone metastasis” OR “bone metastases”) AND (“Radiotherapy”[Mesh] OR “Radiation”[Mesh] OR “Radiotherapy, Conformal”[Mesh] OR “Radiosurgery”[Mesh] OR “Radiotherapy, Intensity-Modulated”[Mesh] OR “radiotherapy” OR “radiation”) AND (“normal tissue” OR “organs at risk” OR “OAR”))

Filters: Publication date from 2010/01/01 to 2020/03/23, Humans, English, Adult: 19+ years

What clinical scenarios can be classified as “complex” and warrant review by a multidisciplinary team?

((“bone metastasis” OR “bone metastases”) AND (“Radiotherapy”[Mesh] OR “Radiation”[Mesh] OR “Radiotherapy, Conformal”[Mesh] OR “Radiosurgery”[Mesh] OR “Radiotherapy, Intensity-Modulated”[Mesh] OR “radiotherapy” OR “radiation”) AND (“complicated” OR “uncomplicated” OR “complex” OR “re-treatment” OR “retreatment” OR “re-irradiation”))

Filters: Publication date from 2010/01/01 to 2020/03/23, Humans, English, Adult: 19+ years

What are important considerations for re-irradiation of non-spine bone mets? (i.e. composite dose, dose constraints, dose de-escalation)

((“bone metastasis” OR “bone metastases”) AND (“Radiotherapy”[Mesh] OR “Radiation”[Mesh] OR “Radiotherapy, Conformal”[Mesh] OR “Radiosurgery”[Mesh] OR “Radiotherapy, Intensity-Modulated”[Mesh] OR “radiotherapy” OR “radiation”) AND (“fractionation” OR “fraction” OR “dose” OR “single” OR “hypofractionated”) AND (“Retreatment”[Mesh] OR “Re-irradiation”[Mesh] OR “re-treatment” OR “retreatment” OR “re-irradiation”))

Filters: Publication date from 2010/01/01 to 2020/03/23, Humans, English, Adult: 19+ years

When should a patient be referred for surgical fixation/stabilization?

((“bone metastasis” OR “bone metastases”) AND (“Surgical Procedures, Operative”[Mesh] OR “surgery” OR “fixation” OR “stabilization”) AND ("trial" or "randomized"))

Filters: Publication date from 2010/01/01 to 2020/04/14, Humans, English, Adult: 19+ years

What is the preferred approach to radiotherapy in the setting of stabilization surgery?

((“bone metastasis” OR “bone metastases”) AND (“Radiotherapy”[Mesh] OR “Radiation”[Mesh] OR “Radiotherapy, Conformal”[Mesh] OR “Radiosurgery”[Mesh] OR “Radiotherapy, Intensity-Modulated”[Mesh] OR “radiotherapy” OR “radiation”) AND (“Surgical Procedures, Operative”[Mesh] OR “surgery” OR “fixation” OR “stabilization” OR “Kyphoplasty”[Mesh] OR “Vertebroplasty”[Mesh] OR “kyphoplasty” OR “vertebroplasty” OR “interventional radiology”) AND ("trial" OR "randomized"))

Filters: Publication date from 2010/01/01 to 2020/03/23, Humans, English, Adult: 19+ years

When should image-guided ablation (or other percutaneous techniques like cementoplasty) be considered for non-spine bone metastases?

((“bone metastasis” OR “bone metastases”) AND (“radiofrequency ablation” OR “ablation” OR “interventional radiology”))

Filters: Publication date from 2010/01/01 to 2020/03/23, Humans, English, Adult: 19+ years

What role do bisphosphonates play in the management of non-spine bone metastases?

((“bone metastasis” OR “bone metastases”) AND (“Diphosphonates”[Mesh] OR “bisphosphonates”) AND ("trial" OR "randomized"))

Filters: Publication date from 2010/01/01 to 2020/03/23, Humans, English, Adult: 19+ years

What systemic therapies should be held during radiation for metastatic disease and for how long?

((“bone metastasis” OR “bone metastases”) AND (“Radiotherapy”[Mesh] OR “Radiation”[Mesh] OR “Radiotherapy, Conformal”[Mesh] OR “Radiosurgery”[Mesh] OR “Radiotherapy, Intensity-Modulated”[Mesh] OR “radiotherapy” OR “radiation”) AND (“Antineoplastic Agents”[Mesh] OR “Chemotherapy, Adjuvant”[Mesh] OR “systemic therapy” OR “chemotherapy”) AND ("trial" OR "randomized"))

Filters: Publication date from 2010/01/01 to 2020/03/23, Humans, English, Adult: 19+ years
